# In Antisynthetase Syndrome, ACPA Are Associated With Severe and Erosive Arthritis: An Overlapping Rheumatoid Arthritis and Antisynthetase Syndrome

**DOI:** 10.1097/MD.0000000000000523

**Published:** 2015-05-22

**Authors:** Alain Meyer, Guillaume Lefevre, Guillaume Bierry, Aurélie Duval, Sébastien Ottaviani, Olivier Meyer, Anne Tournadre, Benoit Le Goff, Laurent Messer, Anne Laure Buchdahl, Michel De Bandt, Christophe Deligny, Matthieu Dubois, Pascal Coquerelle, Géraldine Falgarone, René-Marc Flipo, Alexis Mathian, Bernard Geny, Zahir Amoura, Olivier Benveniste, Eric Hachulla, Jean Sibilia, Baptiste Hervier

**Affiliations:** From the Service de Rhumatologie—Centre de Référence des Maladies Autoimmunes et Systémiques Rares (AMe, JS), Hôpitaux Universitaires de Strasbourg, Fédération de Médecine Translationnelle; Service de Physiologie et d’Explorations Fonctionnelles (AM, BG), Centre Hospitalo-Universitaire de Strasbourg; EA 3072 (AM, BG), “Mitochondrie, Stress Oxydant et Protection Musculaire”, Faculté de Médecine, Université de Strasbourg et Fédération de Médecine Translationnelle (FMTS), Strasbourg; Service de Médecine Interne—Centre de Référence des Maladies Autoimmunes et Systémiques Rares (GL, EH), CHRU Lille, Université Lille 2, Lille; Service de Radiologie (GB), Hôpitaux Universitaires de Strasbourg, Strasbourg; Service de Rhumatologie (AD), Hôpital Général de Dôle, Dôle; Service de Rhumatologie (SO, OM), Hôpital Bichat, APHP, Paris; Service de Rhumatologie (AT), Hôpitaux Universitaires de Clermont-Ferrand, Clermont-Ferrand; Service de Rhumatologie (BLG), Hôpitaux Universitaires de Nantes, Nantes; Service de Rhumatologie (LM), Hôpital Général de Colmar, Colmar; Service de Médecine Interne (ALB), Hôpital Général de Douai, Douai; Service de Rhumatologie (MDB); Service de Médecine Interne (CD), Hôpitaux Universitaire de Fort de France, Fort de France; Service de Pneumologie et Réanimation Médicale (MD), Hôpital Pitié-Salpêtrière, APHP, Paris; Service de Rhumatologie et Néphrologie (PC), Centre Hospitalier de Béthune, Béthune; Department de Rhumatologie (GF), Assistance Publique-Hôpitaux de Paris, Groupe Hospitalier Avicenne-Jean Verdier-René Muret; INSERM UMR1125 (GF); Sorbonne Paris Cité-Université Paris 13 (GF), Bobigny; Service de Rhumatologie Hôpitaux Universitaires de Lille (RMF), Université de Lille 2, Lille; Service de médecine interne 2, Centre de Référence National pour le Lupus et le Syndrome des Antiphospholipides, institut E3M, Hôpital Pitié Salpêtrière, APHP, Paris, F-75013 (AMa, ZA) ; Département de médecine interne et immunologie clinique, Centre de Référence Maladies Neuro-Musculaires – DHU i2B, Hôpital Pitié- Salpêtrière, APHP, Paris, F-75013 (BH, OB) INSERM UMR-S 1135 (BH, AMa, ZA) & INSERM UMRS974 (BH, OB) Sorbonne Universités, UPMC Univ Paris 06, France.

## Abstract

Anticitrullinated peptide/protein antibodies (ACPA), which are highly specific for rheumatoid arthritis (RA), may be found in some patients with other systemic autoimmune diseases. The clinical significance of ACPA in patients with antisynthetase syndrome (ASS), a systemic disease characterized by the association of myositis, interstitial lung disease, polyarthralgia, and/or polyarthritis, has not yet been evaluated with regard to phenotype, prognosis, and response to treatment. ACPA-positive ASS patients were first identified among a French multicenter registry of patients with ASS. Additionally, all French rheumatology and internal medicine practitioners registered on the Club Rhumatismes et Inflammation web site were asked to report their observations of ASS patients with ACPA. The 17 collected patients were retrospectively studied using a standardized questionnaire and compared with 34 unselected ACPA-negative ASS patients in a case–control study. All ACPA-positive ASS patients suffered from arthritis versus 41% in the control group (*P* < 0.0001). The number of swollen joints was significantly higher (7.0 ± 5.0 vs 2.9 ± 3.9, *P* < 0.005), with a distribution resembling that of RA. Radiographic damages were also more frequent in ACPA-positive ASS patients (87% vs 11%, *P* < 0.0001). Aside from a significantly higher transfer factor for carbon monoxide in ACPA–ASS patients, lung, muscle, and skin involvements had similar incidences, patterns, and severity in both groups. Although Nonbiologic treatments were similarly used in both groups, ACPA-positive patients received biologics more frequently (59% vs 12%, *P* < 0.0008), mostly due to refractory arthritis (n = 9). Eight patients received anti-Cluster of differentiation 20 (CD20) monoclonal antibodies (mAbs) with good efficacy and tolerance, whereas 2 of the 5 patients treated with antitumor necrosis factor drugs had worsened myositis and/or interstitial lung disease. After a >7-year mean follow-up, extra-articular outcomes and survival were not different. ACPA-positive ASS patients showed an overlapping RA–ASS syndrome, were at high risk of refractory erosive arthritis, and might experience ASS flare when treated with antitumor necrosis factor drugs. In contrast, other biologics such as anti-CD20 mAb were effective in this context, without worsening systemic involvements.

## INTRODUCTION

Numerous studies have confirmed that anticitrullinated peptide/protein antibodies (ACPAs) are both highly sensitive and specific for rheumatoid arthritis (RA).^1^ This has recently led to their inclusion in the serological criteria for the classification of RA.^2^ In this disease, ACPAs also have a high prognostic significance as they are associated with higher disease activity,^3^ radiographic progression,^4^ and poorer response to therapy.^5^

However, despite their high specificity, ACPA may be found in some patients with other systemic autoimmune diseases, including systemic sclerosis^6–8^ and systemic lupus.^9–12^ Importantly, in these settings, ACPA positivity has been linked to bone erosions resembling RA. For example, in systemic lupus, ACPA positivity is associated with “rhupus” in which the signs and symptoms of RA prevail.^13,14^

The occurrence of ACPA in antisynthetase syndrome (ASS) has been documented in only a few case reports^15–21^ or series involving <10 patients.^22^ ASS is also a systemic autoimmune connective tissue disease, characterized by the association of interstitial lung disease (ILD), inflammatory myopathy, Raynaud phenomenon, and/or mechanic's hands with the presence of antiaminoacyl-transfer RNA synthetase (anti-ARS) antibodies. Arthritis and/or arthralgia are present in up to 90% of these patients,^23–25^ and joint disease is the initial symptom in 21% to 32% of patients with ASS.^19,21^ Subluxation arthropathy has been described in 19% of anti-Jo1–ASS patients,^26,27^ whereas joint damages are less common, ranging generally from 0% to 8%.^26–29^

In light of the above, we retrospectively studied cases of patients displaying ACPA in the context of ASS with the aim of assessing the clinical significance of this association with regard to phenotype, prognosis, and response to treatment.

## METHODS

### Patients

In this case–control retrospective study, patients with ACPA (n = 7) were first identified among a French 9-center registry of 284 patients with ASS.^25^

Additionally, all French rheumatology and internal medicine practitioners registered on the Club Rhumatismes et Inflammation web site (representing >1400 physicians) were contacted by 2 successive electronic newsletters and asked to report their observations of ASS patients with ACPA who met the following inclusion criteria: 2 successive positive tests for anti-ARS, including LUMINEX-100 system (Luminex, Austin, TX), ENA-LISA-kit (BioMedical Diagnostics, Marne-la-Vallée, France), or IMMUNO-DOT (Euroimmun AG, Lübeck, Germany; DiaSorin, Saluggia, Italy); clinical involvement in accordance with ASS, including pulmonary, muscle, dermatological, or rheumatic involvements;^23^ ACPA positivity using anti-cyclic citrullinated peptide 2 (CCP2) assays, including BioPlex 2200 System (Bio-Rad Laboratories, Hercules, CA), Immunoscan-CCP Plus (Eurodiagnostica, the Netherlands, Arnhem), Quantalite CCP2 (Inova, San Diego, CA), and Elecsys anti-CCP (Roche, Meylan, France). Among 13 reported patients, 3 did not meet the inclusion criteria: 2 patients had solely rheumatic involvement and 1 had solely pulmonary involvement. Patients were included from May 1, 2012 to May 1, 2014.

The 17 ACPA-positive ASS patients included in the study were compared with 34 unselected ASS patients from the French 9-center registry matched for age, sex, and follow-up, fulfilling inclusion criteria,^1,2^ whereas being tested negative for ACPA.

### Data Collection

Demographic information, comorbidities, clinical history of ASS, imaging findings (including hand and foot radiographs, high-resolution computed tomography (HRCT)] thoracic scans, pulmonary function tests, biological data, and detailed medical treatment were collected. Data collection was compiled by A.M., G.L., and B.H. using the same standardized form.

### Definitions

ACPA were considered positive when superior to the upper limit of normal (ULN) for the laboratory of immunology.^2^

Joint involvement included arthralgia and/or arthritis. Severity was assessed through higher recorded values of tender joint count (TJC), swollen joint count (SJC), and appearance of radiographic damage. Radiographic damage was defined by the presence of bone erosion and/or joint narrowing on standard radiographs of the hand and/or the foot. All radiographs were examined by experienced radiologists and rheumatologists. Hand radiographs were also retrospectively examined blinded to ACPA status by A.M., G.B., and J.S., and radiographic damages were scored using the Sharp method^30^ with additional scoring of the distal interphalangeal joints (IPJs) of the hands. Consensus was always obtained.

Pulmonary involvement was defined by the presence of an ILD on HRCT, confirmed in most cases by abnormal pulmonary function tests (forced vital capacity [FVC] < 70% and/or diffusing capacity of the lung for carbon monoxide [DLCO] < 70%).

Muscle involvement^31^ was defined either by the occurrence of myalgia, muscle weakness (Medical Research Council 5 sum score ≤4 for at least ≥1 muscle group evaluated by manual muscle testing [MMT]), along with increased creatine kinase (CK) level >2 times the normal level, myopathic changes on electromyography, or typical features in muscle biopsies (assessed by different pathologists experienced in muscle histopathology).

Outcome was assessed for each patient during the follow-up period. Changes ≥30% in TJC and/or SJC were considered to be significant in defining aggravation, stability, and improvement of joint disease. Pulmonary aggravation, stability, and improvement were defined as changes in pulmonary function tests >15% for DLCO or >10% for FVC, in accordance with the international consensus statement of the American Thoracic Society on idiopathic pulmonary fibrosis,^26^ concordant with evolution in dyspnea according to New York Heart Association stages, and/or to HRCT results. Muscle aggravation, stability, and improvement, respectively, corresponded to a decrease, stability, or increase in MMT or a ≥2-fold modification in CK levels, as compared with the time of ASS diagnosis.

### Statistical Analysis

Quantitative data were described as mean ± SD (unless specified otherwise). Mann–Whitney and Fisher tests were used for comparison of continuous and categorical variables, respectively. The Kaplan–Meyer method and log rank tests were used to compare survival between groups. Only a *P* value <0.05 was considered significant. All analyses were performed using GraphPad Prism 5 software (San Diego, CA).

## RESULTS

Polyarthralgia and/or polyarthritis were the first symptoms to appear in the majority of ACPA-positive ASS patients (n = 88%), whereas myositis and/or ILD were present at onset in 41% and 35% of patients, respectively. ACPA were identified 3 months (range 0–132) after the first symptoms whereas antisynthetase autoantibodies were found 2 months (range 0–145) after disease onset. Initial diagnoses were RA (n = 6), ASS (n = 5), dermatomyositis (DM) (n = 3), polymyositis (n = 1), and RA–ASS overlapping syndrome (n = 2). Clinical characteristics of the 17 ACPA–ASS patients are shown in Table 1.

**TABLE 1:**
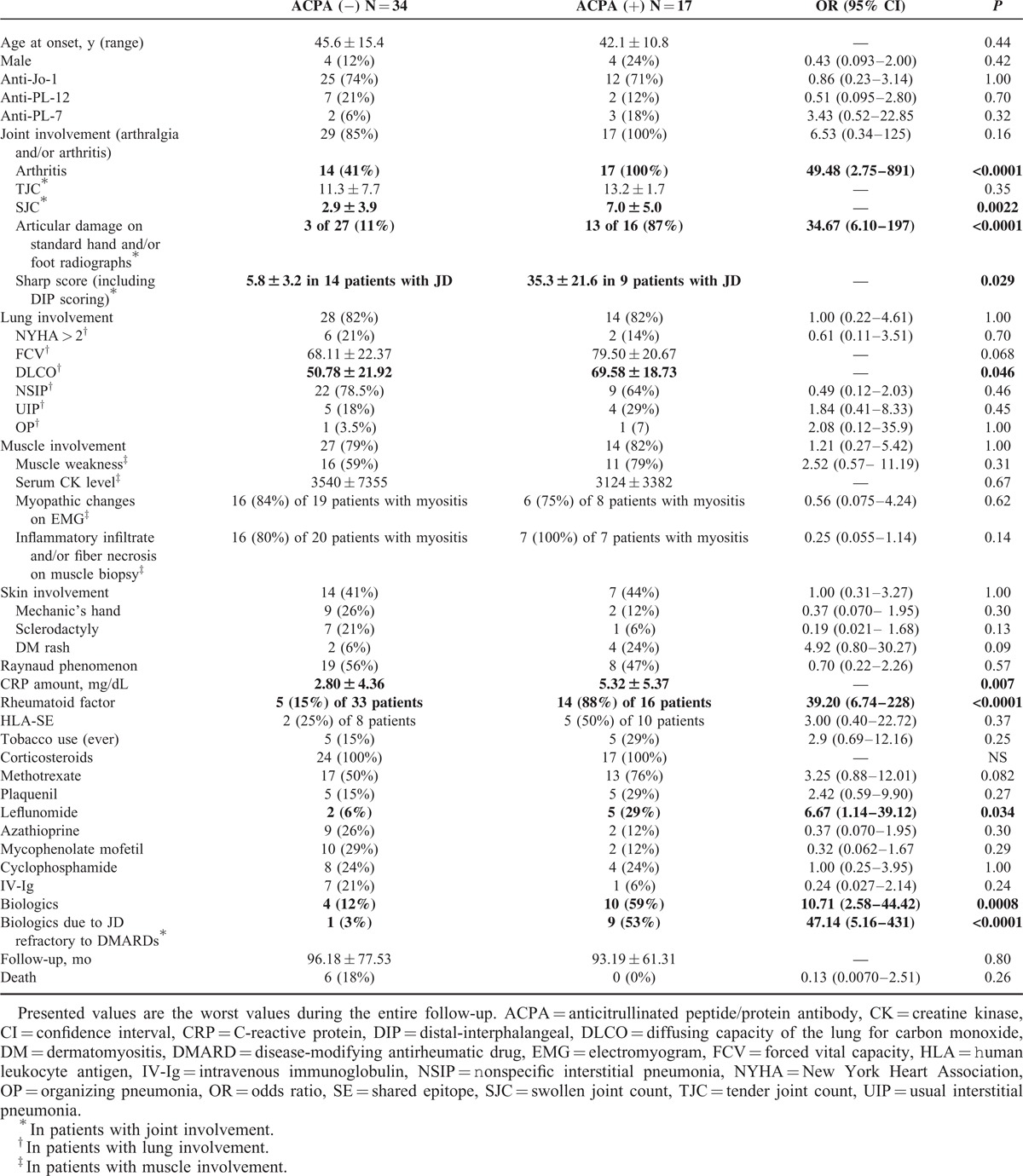
Comparison of Clinical Manifestations Between ACPA-Positive and ACPA-Negative SAS Patients

### Demographic Characteristics

Among the 17 ACPA-positive ASS patients, there were 4 men and 13 women, with a mean age at onset of 45.6 ± 15.4 years (Table 1). There were no significant differences in terms of sex or age at onset between ACPA-positive ASS patients and the control group. Of note, the proportion of smokers was not significantly higher in ACPA-positive ASS patients (29% vs 15%, *P* = 0.25).

### Clinical Characteristic of ACPA-Positive ASS Patients

Despite a similar incidence of joint involvement in both groups, all ACPA-positive ASS patients suffered from arthritis versus 14 patients (41%) in ACPA-negative ASS patients, resulting in an odds ratio (OR) for arthritis of 49.5, 95% confidence interval (CI) 2.8–891, and *P* < 0.0001 (Table 1). When solely considering patients with joint involvement, the number of swollen joints was significantly higher in the ACPA-positive ASS group (2.9 ± 3.9 vs 7.0 ± 5.0, *P* = 0.0022). Distribution of arthritis (n = 16/17) was always symmetric and mainly involved metacarpophalangeal (MCP) joints (n = 14), wrists (n = 10), and proximal IPJ of both hands (n = 8). Knees (n = 7), ankles (n = 4), elbows (n = 4), and distal IPJ (n = 1) were less commonly involved. There was no difference in the pattern of joint involvement between ACPA-positive and ACPA-negative patients.

Although ILD affected 82% of the patients in both groups, ASS patients with ACPA tended to display higher FVC (68.11 ± 22.37 vs 79.50 ± 20.67) and had higher DLCO compared with the ACPA-negative group (50.78 ± 21.98 vs 69.58 ± 18.73, *P* = 0.046). The distribution of the different ILD patterns, according to international consensus,^32^ was similar in both groups. No patient exhibited pulmonary rheumatoid nodules.

Patients from both groups were equally affected by myositis (about 80%, *P* = 1.00). Furthermore, there were no differences with regard to occurrence of muscle weakness (59% vs 79%, *P* = 0.31), CK amount (3540 ± 7355 vs 3124 ± 3382, *P* = 0.67), and frequency of myopathic changes recorded on electromyogram (84% vs 75%, *P* = 0.62). When performed (n = 7), muscle biopsy features in patients with ACPA included inflammatory infiltrate (endomysial n = 3, perimysial n = 2, and perivascular n = 2), muscle fiber necrosis (n = 4), and perifascicular atrophy (n = 2), which did not differ from the ACPA-negative group (data not shown).

Patients with ACPA also exhibited Raynaud phenomenon (47%), DM rash (24%), mechanic's hands (12%), and/or sclerodactyly (6%), in similar proportions to the control ASS group.

### Radiographic Characteristics of ACPA-Positive ASS Patients

**FIGURE 1:**
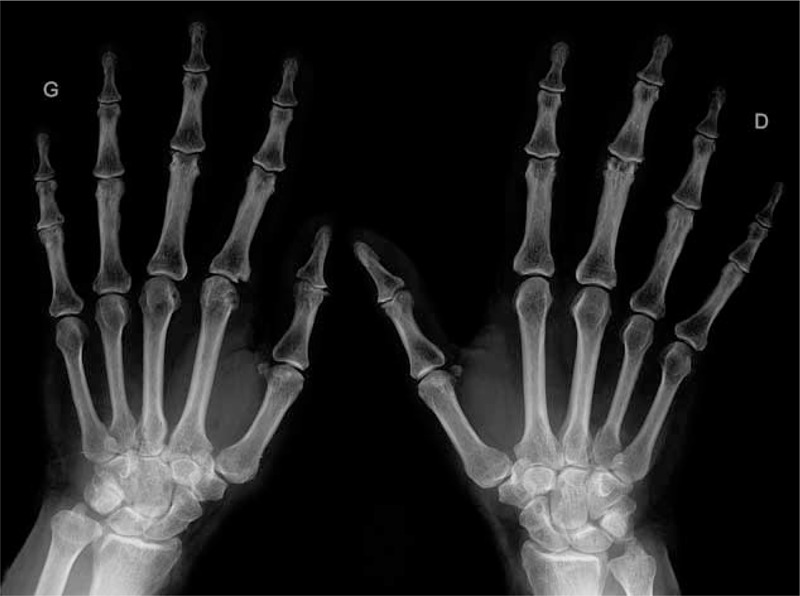
Representative hand radiographs in ASS patients with ACPA. ACPA = anticitrullinated peptide/protein antibody, ASS = antisynthetase syndrome.

Radiographic damages were more frequent in ACPA–ASS patients (13/16 [87%]) vs 3/27 (11%) patients with joint disease (OR 34.67, 95% CI 6.1–197.0, *P* < 0.0001) (Table 1 and Figure 1). In ACPA–ASS patients, bone erosion and/or joint narrowing were observed 40.8 ± 19.8 months after joint disease onset, and involved MCP joints (n = 9), metatarsophalangeal joints (n = 7), wrist (n = 4), PIP (proximal interphalangeal) joints (n = 4), and/or DIP (distal interphalangeal) (n = 1). In contrast, in the 3 ACPA-negative patients with radiographic damages, only joint narrowing with no erosion was observed, involving wrists (n = 3), MCP (n = 2), PIP (n = 1), and/or DIP (n = 1) joints, all of which tended to be diagnosed later during follow-up: median 63 months, range 106–118 (*P* = 0.11).

Sharp score (including DIP joints) assessed blindly the ACPA status after examination of the last available hand radiographs, and was higher in ACPA-positive patients (n = 9/17, 53%) compared with ACPA-negative patients (n = 14/34, 41%): 35.3 ± 21.6 vs 5.8 ± 3.2, *P* < 0.05.

### Biological Characteristics

Distribution of anti-ARS specificities was similar between the 2 groups, anti-Jo1 being the most common (always above 70% of patients, Table 1). Median ACPA-titer in ACPA–ASS patients was 200 UI/L, range 33–7742. Rheumatoid factor was found in 14/16 patients (88%) versus 5/33 patients (15%) in the control group (OR 39.20, 95% CI 6.74–228, *P* < 0.0001), and ACPA-positive ASS patients exhibited higher CRP amount compared with ACPA-negative ASS patients (5.32 mg/dL ± 5.37 vs 2.80 ± 4.36, *P* = 0.007). Among 10 ASS patients with ACPA tested for human leukocyte antigen (HLA) haplotypes, 5 (50%) were HLA-shared epitope (SE) positive (HLA-DR4 [n = 4] or HLA-DR10 [n = 1], heterozygous in all cases), although this occurrence was similar to the ACPA-negative group (25%, *P* = 0.37).

### Treatments

Mean follow-up after onset of symptoms was 96.2 ± 77.5 and 93.2 ± 61.3 months in ACPA-negative and ACPA-positive groups, respectively (*P* = 0.80) (Table 1).

All of the patients with ACPA received prednisone (n = 17) with a maximum dosage during follow-up (46.8 ± 17.8 mg/d) comparatively with the ACPA-negative patients (42.2 ± 19.5 mg/d (n = 25), *P* = 0.35). Additionally, at least 1 additional immunomodulating therapy was used, including methotrexate (n = 13), hydroxychloroquine (n = 5), leflunomide (n = 5), sulfasalazine (n = 2), azathioprine (n = 2), mycophenolate mofetil (n = 2), cyclophosphamide (n = 4), and/or intravenous immunoglobulin (n = 1). At the end of the follow-up period, at least 2 nonbiologic drugs were used in both groups (*P* = 0.53). With the exception of leflunomide, which was more frequently given in ACPA-positive ASS patients (*P* = 0.007), there was no qualitative difference in nonbiologic treatments received between both groups. Biologics, including rituximab (n = 8), infliximab (n = 4), etanercept (n = 1), adalimumab (n = 1), tocilizumab (n = 1), and/or abatacept (n = 1), were more frequently used in ACPA-positive patients (59% vs 12%, *P* < 0.0008). Of note, in 9/10 ACPA–ASSpatients treated with biologics, the indication for initiatingsuch treatment was refractory arthritis.

### Biologics Tolerance and Efficacy

In 10 patients, a total of 16 biologic courses were used that included anti-Cluster of differentiation 20 (CD20) monoclonal antibody (mAb) (n = 8), antitumor necrosis factor (TNF) drugs (n = 6 courses in 5 patients, 1 patient receiving infliximab followed by adalimumab), anti-interleukin-6 receptor mAb (n = 1), and inhibitor of T-cell costimulation (n = 1) (Table 2).

**TABLE 2:**
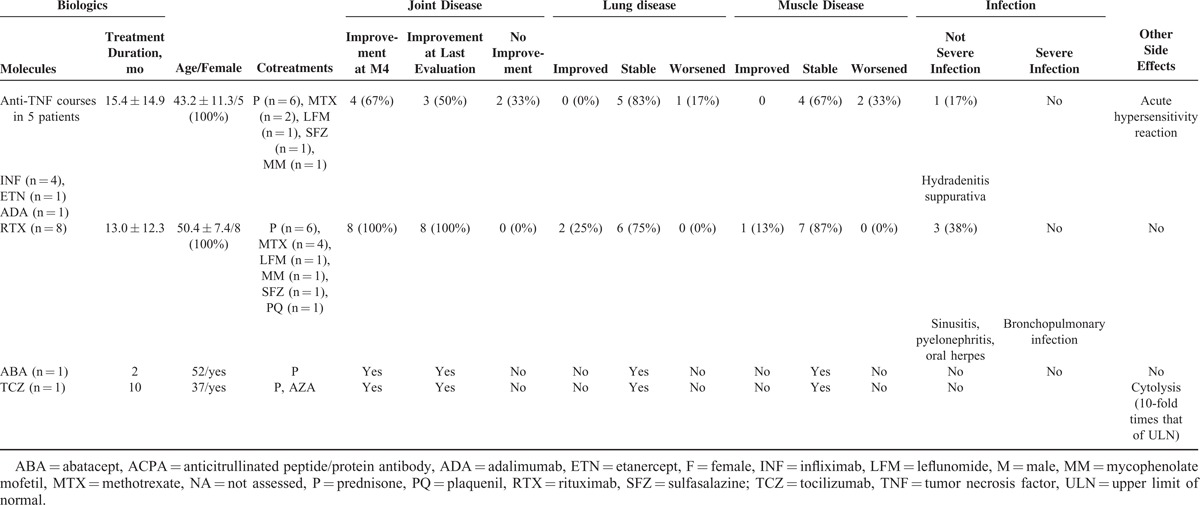
Tolerance and Efficacy of Biologics in 10 ACPA-SAS Patients

Anti-CD20 mAb led to joint efficacy along with improvement and/or stability of extra-articular involvements in all cases (n = 8).

Anti-TNF treatments resulted in joint improvement in 4/6 courses (67%). However, in 2 cases (33%), systemic involvement worsened (myositis n = 2 and/or ILD n = 1).

In patients treated with other biologics (n = 2), both experienced improvement in joint disease, without systemic flare-up of ASS, although they exhibited increases in transaminase levels (up to 10-fold that of ULN, whereas CK amount was normal) and severe bronchopulmonary infection, leading to drug withdrawal.

### Outcomes

**FIGURE 2:**
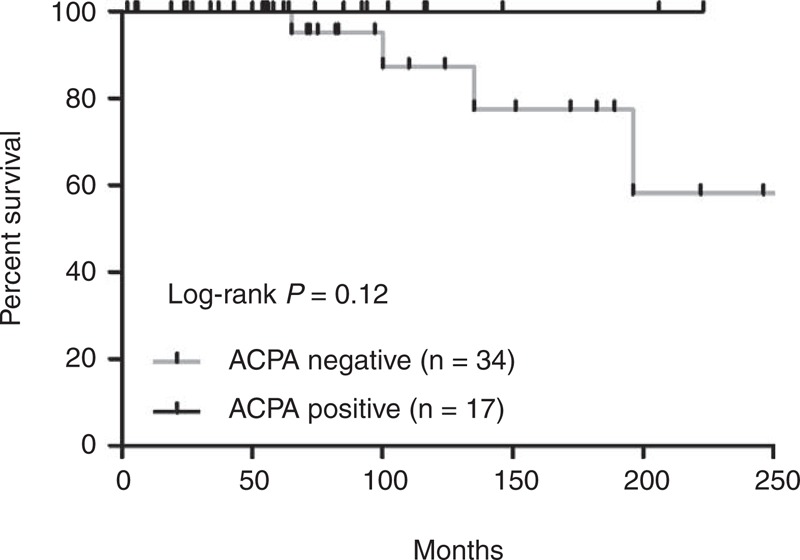
Kaplan–Meyer survival curve (from disease onset) comparing patients with (n = 17) and without (n = 34) ACPA. ACPA = anticitrullinated peptide/protein antibody.

At the last clinical evaluation, joint disease had improved in the majority of patients in both groups (23/25 and 16/17, *P* = 1.0). In the ACPA-negative group, 6 of the 23 patients with lung disease had worsened ILD versus 1 in the ACPA–ASS patients (*P* = 0.27). Myositis had improved in all cases, regardless of ACPA status. Seven ACPA-positive patients (41%) were still under corticosteroid treatment with a dosage ≥10 mg/d versus 8/27 (30%) in the ACPA-negative group (*P* = 0.53). Finally, all patients with ACPA were alive at the end of the follow-up period, whereas 4 patients in the control group had died (*P* = 0.29). Causes of death were ILD (n = 3) or concomitant multiple myeloma (n = 1). As a result, the survival rate was not statistically different between the 2 groups (*P* = 0.15, Figure 2).

## DISCUSSION

This study, which is the first case–control study to assess the clinical significance of ACPA in patients with ASS, suggests that ASS-patients with ACPA have an overlapping RA–ASS syndrome. Indeed, polyarthritis was present in all ACPA-positive patients, involving numerous small joints, with a distribution resembling that of RA, along with higher CRP amount and a positive test for rheumatoid factor. Thus, although all patients displayed ASS manifestations and were tested positive for ARS at least twice, all ACPA-positive patients also fulfilled the recently proposed 2010 American College of Rheumatology diagnostic criteria for RA^2^ versus 19 ACPA-negative patients (*P* = 0.0008). Accordingly, and as also demonstrated in the setting of RA, ACPA positivity in ASS patients was associated with a greater occurrence of disease-modifying antirheumatic drugs (DMARDs)-refractory arthritis as well as a higher incidence of joint damages.

Arthritis, ILD, and Raynaud phenomenon are symptoms encountered in both RA and ASS.^24,25,33,34^ Thus, one third of our ACPA–ASS patients were first diagnosed as having solely RA. However, during the course of the follow-up, all but 2 patients developed at least 1 additional sign of ASS, including typical dermatological signs and/or myositis. Finally, the occurrence of lung, muscle, and skin involvement did not differ between ACPA-positive and ACPA-negative patients. This suggests that RA patients experiencing such systemic symptoms should be tested for anti-ARS as these autoantibodies are associated with ASS even in the setting of RA. A higher DLCO was recorded in ACPA–ASS patients with ILD, which was the only difference in extra-articular involvement between the 2 groups. DLCO has been shown to be a predictor of survival time in ILD patients^32^ including RA–ILD^35^ and myositis–ILD.^36^ Although 4 deaths were recorded in the ACPA-negative group versus none in the ACPA-positive group, difference in survival between the 2 groups did not reach statistical significance despite prolonged follow-up.

ACPA is strongly associated with the HLA-DRB1 SE and with smoking. ACPA–ASS patients tended to be more frequently smokers and HLA-SE positive. However, as the trend did not reach statistical significance (likely due to the limited number of patients herein), the role of these factors in the development of ACPA during ASS remains to be further explored.

The present data extend the findings of the 9 previously described ACPA–ASS patients, reported as separate case reports, in which the presence of ACPA was systematically associated with erosive arthritis^15–20,37^ (Table 3). This is also in keeping with recent findings from Kaneko et al,^22^ which reported that in ASS patients with available hand X-rays, erosions were associated with higher rates of positive ACPA and rheumatoid factors. Among 90 consecutive patients with idiopathic inflammatory myopathy, the 12 patients that were tested positive for ACPA (including only 2 patients with anti-ARS) did not show any clinical or biological differences compared with ACPA-negative patients, except for rheumatoid factor positivity.^21^ This discrepancy with the present findings may be the result of the small number of ACPA–ASS patients, differences in diagnostic test specificity,^38^ and/or limited follow-up.

**TABLE 3:**
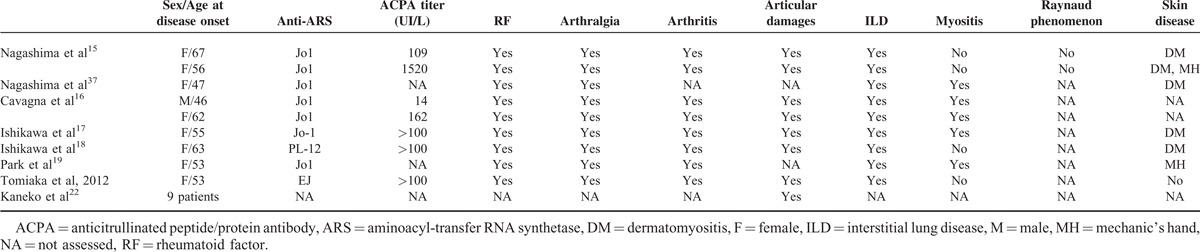
Clinical Characteristics of the 9 ACPA-SAS Patients Previously Reported in Literature

According to the present data and given that structural damages in RA are believed to be largely irreversible albeit preventable by tight control of joint disease, we suggest that ACPA should be searched in ASS patients, especially those with severe polyarthritis and high CRP amount.

In the current series, the ACPA-positive patients experienced more frequently DMARDs refractory. Although there is no consensual therapeutic strategy for such patients in ASS, in RA patients, it is recommended to start biologic DMARDs, and current practice would be to start a TNF inhibitor.^39^ However, it has recently been reported that anti-TNF agents may not be effective in ASS patients and may even trigger myositis and/or ILD in ASS.^17,18^ Indeed, among 6 anti-TNF courses in our series, 1 led to the development of myositis and ILD, whereas another led to worsening myositis. Because Rituximab has demonstrated efficacy in RA^40^ and might be effective in ASS muscle, lung, and joint disease,^41–44^ this drug has frequently been given. No serious adverse event was observed and arthritis improved in all cases along with stability and improvement of extra-articular manifestations, suggesting that this biologic is safe and efficient in ACPA–ASS patients with severe refractory joint diseases.

This study assessed the clinical significance of ACPA retrospectively in a real-life management of ASS patients. ACPA were not tested in all the patients recorded in the multicenter registry, and it is possible that a proportion of ASS patients included in this study were tested for ACPA because they had polyarthralgia and/or polyarthritis. Indeed, compared with the totality of the patients recorded in the registry, ACPA-negative ASS patients included in this study had higher frequency of polyarthralgia (85% vs 63%, *P* < 0.05) and polyarthritis (41% vs 20%, *P* < 0.05).^25^ Thus, our study may actually underestimate the prognostic significance of ACPA statute for joint disease in ASS patients.

In summary, the present series demonstrates that ASS can overlap with RA. The recognition of this setting among patients diagnosed with ASS, but also in patients diagnosed with RA, has important implications for both prognosis and management. ACPA-positive ASS patients display extrarheumatic manifestations similar to ACPA-negative ASS patients and are at high risk of developing erosive arthritis refractory to DMARDs. Conversely, to anti-TNF drugs, anti-CD20 mAb may be effective and well tolerated in these patients, without exacerbating extra-articular involvements.
